# Leukoreduced blood components: Advantages and strategies for its implementation in developing countries

**DOI:** 10.4103/0973-6247.59384

**Published:** 2010-01

**Authors:** R. R. Sharma, Neelam Marwaha

**Affiliations:** *Department of Transfusion Medicine, PGIMER, Chandigarh, India*

**Keywords:** Blood component, developing countries, leukoreduced component

## Abstract

Removal of leucocytes from various blood products has been shown to minimize Febrile nonhemolytic transfusion reactions, HLA alloimmunization, platelet refractoriness in multitransfused patients and prevention of transmission of leukotropic viruses such as EBV and CMV. Rapidly growing size of hemato-oncological patients in our country requiring multiple transfusion of blood and components during the course of their management pose a great challenge to transfusion services to provide them red cell and platelet antigen matched products in alloimmunized subjects. Thus removal of leucocytes below a certain threshold, ≤ 5 × 10^6^ in a blood component certainly helps in prevention of alloimmunization and associated risks in these patients. Currently the best Leucoreduction can be achieved with the help of 3rd and 4th generation leukofilters, both in laboratory and patient bed side, and state of the art apheresis devices. The present article briefly reviews the current literature for pros and cons of leucofilteration and its scope of implementation in the cost constrained settings.

The past fifty years has seen a significant, paradigm shift in the provision of allogenic blood products. Half a century ago, most of the blood transfused was whole blood. However, since the 1960s, whole blood has been separated into its various components such as RBCs, platelets, and plasma. The latter has been further subjected to various manufacturing processes so that individual plasma proteins can be purified and made available to specific patients with specific plasma protein deficiencies.

Until recently, little attention had been paid to the leukocytes present in various blood components. However, it has been shown that the removal of leukocytes from various blood products can minimize the risks[1–6] associated with these contaminating leukocytes; the most common among which are: Nonhemolytic febrile transfusion reactions, human leukocyte antigen (HLA) alloimmunization and platelet refractoriness observed in multi-transfused patients, and transmission of leukotropic viruses. Although the terms, leukoreduction and leukodepletion are used interchangeably in literature, leukoreduction technically implies removal of leukocytes by gross removal method, whereas, leukodepletion connotes removal of leukocytes with the help of certain specific filters or devices.

## Need for Leukoreduction in India

India has a population of one billion and has a huge burden of patient population requiring multiple transfusions. As per the National AIDS Control Organization, there is a requirement of 8.5-9 million units of blood in our country annually, and this includes the existing Thalassemic population requiring regular transfusions and the rapidly growing size of the hemato-oncology patients requiring different types of blood component support. A majority of them become alloimmune to various red blood cell, platelet, and HLA antigens during the course of their transfusion therapy. This leads to various immunohematological problems in managing the blood component support to these patients, especially the platelet transfusion support in refractory patients. Therefore, transfusion of leukoreduced blood components assumes a lot of significance in these patients.

## Historical Aspects

The concept of removal of leukocytes from the blood was introduced by Fleming, as early as 1920. Fleming used a cotton wool plug in a bent glass tube with a constricted limb. Blood was placed above the cotton wool and forced through it with the help of a teat. It was later realized that this model closely resembled the structure of modern depth filters. The work on leucofilteration got a boost subsequent to the accidental observation by Swank in 1961, while working on a blood viscosity model, wherein, on microscopic examination, he found that very high pressure was required to force 2-10 days old acid-citrate-dextrose (ACD)-stored blood through a microfilter, as aggregates of platelets and leukocytes clogged the filter passes. Later, in the 1980s, advancement in technology led to the development of the first generation cellulose acetate filters, with a leukocyte removal efficiency of 98 percent. Although clinically acceptable results were achieved, they had two major limitations. First, they appeared to activate complement C3, with subsequent vasoconstriction and increased capillary permeability. Second, the efficacy of leukocyte removal was strongly dependent on the flow across the filter, so the overall filtration process was slow. The new generation filters with rapid flow and excellent leukocyte removal are discussed later in the text.

## Currently Accepted Standards for Leukoreduced Blood Components

It has been estimated that a freshly collected, whole blood unit contains roughly 10^9^ leukocytes and their concentration continues to decrease with subsequent component processing as shown in Tables 1 and 2.

**Table 1:** Approximate residual number of leukocytes in blood components[7]

Fresh whole blood	10^9^
Red cell concentrate	10^8^-10^9^
Buffy coat-depleted red cells	10^8^
Washed red cell concentrate	10^7^
Frozen deglycerolized red cells	10^6^-10^7^
Platelet concentrate	10^7^-10^8^
Apheresis platelets	10^6^-10^8^
Fresh frozen plasma	< 10^4^

**Table 2:** Current accepted standards for leukodepleted blood components

	Blood component (WB, PRBCs)	RDP for pooling
American Association of Blood Banks (USA)	WB, PRBCs and Aphersis platelet	≤8.3 × 10^5^ WBC/Unit <5 × 10^6^ WBC/Unit (red cell loss not more than 15%)
European Council criteria	<1 × 10^6^ WBC/Unit (Hb > 40/unit)	≤2.0 × 10^5^ WBC/Unit
Director General of Health Services (India) criteria	<5 × 10^6^ WBC/unit (red cell loss not more than 10%)	<8.3 × 10^5^ WBC/Unit

*RDP- Random donor platelets

### In blood Components^6a^

Keeping in view the variability of leukocyte numbers in the component and[8] the leukoreduction method, the leukocyte content in a blood component unit should be less than 5 × 10^6^/unit after leukoreduction (3 log reduction 99.9%) with a minimum of 85 percent red cell recovery in 95 percent of the units tested, as per the standards of the American Association of Blood Banks.[9] The European council guidelines are a little more stringent in terms of residual leukocyte content and require it to be less than 1 × 10^6^/unit. The current accepted standards are briefly summarized in Table 2.

### Modes of leukocyte depletion

The original leukocyte depletion filter contained sterile cotton wool as a filtering agent and was designed by Diepenhorst who published his work in 1972.[10] Subsequently cellulose acetate filters were discovered and found to be more suitable. Other methods included red cell washing, centrifugation and buffy coat removal, freezing and deglycerolization of red cells, and blood component collection through apheresis technology.[11] Of all these methods, leukoreduction by leukofilters (third generation) and components collected through apheresis devices meet the current standards of leukocyte depletion, that is, < 5 × 10^6^ WBC/unit of blood component,[12] whereas, other methods achieve leukocyte depletion to a variable extent, as follows:

- Centrifugation and buffy coat removal — 10^8^ WBCs (1 log leukodepletion)
- Washed red cell concentrate — 10^7^ WBCs (1-2 log leukodepletion)
- Frozen deglycerolized red cells — 10^6^-10^7^ (2-3 log leukodepletion

Thus, for efficient leukoreduction of blood components and to meet the accepted standards, leukocyte filters and leucacytapheresis devices (apheresis machines) are the best.

### Leukofiltration

Current generation of leuko filters (third and fourth) have excellent leukocyte removal efficiency (99.99%) as compared to the first and second generation filters (90-96%). Presently we have depth and screen-type filters. Depth filter (non woven) has filter material in the form of compressed wool fibers arranged in an irregular fashion, whereas, screen filters (woven type) have fibers arranged in multiple layers in a regular fashion.

The primary mechanism[13] of leukocyte removal is the charge-based adhesion of negatively charged leukocytes to the filter material by Vander Waals and electrostatic forces. This adhesion is an active process and has the advantage of larger pore size, by which a subsequent higher flow rate is possible in the filter. The surface charge of the filters can be modified by coating the filter material with methacrylate polymers, to create a stronger positive charge and hence increase the efficiency of the filter.

### Timing of leukofiltration

Leukofilteration of blood components can be done either at the time of collection and processing, post processing (within the blood bank), or by the side of the patient (post storage). Each of them has their own advantages and disadvantages. However, pre-storage leukoreduction is currently the most widely accepted mode. In the Western world the advantages of pre-storage over post-storage leukoreduction are as follows:

- It eliminates the scope of inflammatory (interleukin-1, interleukin-6, tumor necrosis factor) cytokine accumulation due to leukocytes, during storage, and hence, is quite efficient in the prevention of febrile non-hemolytic transfusion reactions.[14–16]
- It also minimizes the risk of HLA-alloimmunization in multitransfused patients, as it removes the intact leukocytes as against filtration, at the bedside, where leukocyte fragments after storage can pass through filters and alloimmunize the recipient against donor antigens.[17,18]
- Pre-storage leukofiltration can also minimize the risk of leukotropic virus transmission as leukocytes disintegrate and release the intracellular organisms after 72 hours of storage in blood components.[19,20]
- It is always easier to perform leukocyte quality control in the laboratory rather than by the patient's bedside. Hence during pre-storage leukoreduction, blood components can be thoroughly studied and evaluated for quality control, and various factors affecting the process of leukofiltration modified accordingly.[21,22]

At present these factors favor pre-storage leukoreduction, either in the form of universal leukoreduction for all the patients or as a selective protocol for a special group of patients. The major drawback with universal leukoreduction is the cost involved; however, selective leukoreduction has its inherent issues of inventory management, as it is quite difficult to predict the requirement of leukoreduced blood components at the time of component preparation.

In contrast, bedside leukoreduction can be done selectively for the patient groups recommended for leukoreduced blood components; however, it has not been found[23–25] to be as effective as pre-storage leukoreduction in decreasing the leukocyte's adverse effects. Thus, the consensus is more in the favor of pre-storage leukoreduction, and the policy of universal leukoreduction of blood components has been adopted in various European countries and some states of the United States of America, whereas, selective bedside leukoreduction is more prevalent in other parts of the world.

### Low leucacytapheresis devices

Blood components collected with the help of the current generation low leucacytapheresis devices are generally 3 logs reduced and hence require no further filtration to meet the standards of leukoreduced blood components. These devices achieve a high degree of separation between the donor platelets and leukocytes as a result of several design principles. Currently the US, FDA-approved equipments for leukoreduced blood components are Cobe Spectra LRS from Gambro, CS 3000 plus, Amicus from Baxter, and MCS plus from Hemonetics and from Fresenius.

## Process Control of Leukoreduced Components

To achieve the expected clinical benefits of providing leukoreduced blood and components, the transfusion centers need to control the overall process so that the prepared product retains an adequate amount of therapeutic blood cells intended for the transfusion. Guidelines for the statistical process control of leukoreduced blood components have been published. [26–28] 10^6^ WBCs per unit translates into 3.3 WBCs/μl, hence the enumeration of residual WBCs in the leukoreduced blood components is a challenging task. The traditional automated cell counters do not give accurate results at leukocyte concentrates less than 100 WBCs/μl. Several[29–31] techniques have been listed in literature such as the cytospin method, Nageotte chamber counting, flow cytometric counting, volumetric cytometry, and counting based on polymerase chain reaction.

Out of all the leukocyte enumeration techniques, the Nageotte chamber counting technique has been found to be quite practical and cost effective in a blood center setting, whereas, other techniques are cumbersome and labor intensive, with more of a research interest.

## Currently Accepted Clinical Indications for Leukoreduced Blood Components

The clinical indications for the use of leukocyte reduced blood components continue to evolve [Table 3]. Need for higher performance filters, the perception of clinical benefits, and the increasing use of pre-storage leukoreduction has resulted in a greater use of leukoreduced components. There has been a considerable debate on whether all cellular blood components should undergo leukoreduction or a selective leukoreduction protocol should be followed. However, the improved outcome related to leukoreduction can be divided into two — whether each benefit has been proven, by evidence-based guidelines, to be clinically relevant, or those that are not proven beneficial or are considered only theoretically relevant.

**Table 3:** Clinical benefits of leukocyte reduction[32]

Proven benefits relevant clinically
Reduced frequency and severity of FNHTRs
Reduced risk of CMV transmission
Reduced risk of HLA-alloimmunization and platelet refractoriness
Probably clinically relevant
Reduced infectious risk associated with immunomodulation (TRIM)
Reduced organ dysfunction and mortality
Reduced direct risk of transfusion-transmissible bacteria
Unproven clinically
Avoidance of vCJD transmission
Avoidance of HTLV I/II, EBV etc.
Reduced risk of GVHD
Reduced risk of TRAL

Thus, the reduction in the number of leukocytes, in allogenic blood products has been proven to be clinically relevant in the following:

- Reducing the frequency and severity of Febrile Non-Hemolytic Transfusion Reactions (FNHTRs).
- Reducing the risk of cytomegalovirus (CMV) transmission.
- Reducing the risk of HLA-alloimmunization and platelet-refractoriness.

- Until recently, the role of leukoreduction in ameliorating the rate of non-hemolytic febrile transfusion reactions (NHFTRs) had not been established, however, there have been several recent studies[33,34] which showed that pre-storage leukoreduction was associated with significant reduction in the NHFTR rate following RBC and (0.35 to 0.18%) platelet transfusions (from 0.4 to 21.3% to 0.11 to 12.3%), thereby causing a decline of up to 50 percent in red cells and 30 percent in platelet transfusion NHFTRs.
- There are a variety of transfusion situations, particularly in immunosuppressed patients who have an increased risk of transfusion-acquired CMV infection. These high-risk recipients include: Low birth weight infants, some oncology patients, and allogenic bone marrow transplant recipients. The prevalence of post-transfusion CMV risk by employing any prevention technology may be as high as 30 percent per patient, depending upon the frequency of allogenic blood product transfusions. Thus transfusion of blood from CMV sero-negative donors and leukoreduced blood components to these patients, has been shown to decrease this risk to 1.3[35] and 2.5 percent,[36] respectively. Hence pre-storage leukoreduction can be considered equivalent to serologically negative CMV allogenic transfusion. However, very high-risk patients, such as allogenic bone marrow transplant recipients, should ideally receive leukoreduced blood products from CMV sero-negative donors, according to a recent conference on the subject.
- This issue has been debated in literature[1–4,16,17] for over the last two decades and major trials have clearly shown that the relative risk of HLA alloimmunization can be reduced considerably through the use of leukoreduced blood products. This is especially helpful in multiply transfused hemato-oncology patients, who are prospective bone marrow transplant and solid organ transplant candidates. In addition, prevention of HLA alloimmunization also helps in minimizing the incidence of platelet refractoriness in these patients.

### Clinical indications likely to benefit from leukoreduction

- Reduced infectious risk associated with immunomodulation (TRIM)
- Reduced organ dysfunction and mortality
- Reduced direct risk of transfusion-transmissible bacteria

### Risks associated with transfusion associated immunomodulation (TRIM)

Transfusion-related immunomodulation has been shown to improve renal allograft survival in renal transplant recipients. There is enough evidence in literature,[37–39] which clearly shows that the use of non-leukoreduced blood improves renal allograft survival. In addition, experimental studies[40] in animals have shown that allogenic blood transfusion promoted tumor growth and its recurrence; however, the data in humans[41] is quite confusing, where some studies clearly indicate that allogenic blood transfusion has a significant adverse effect on tumor growth and recurrence, whereas, others refute such an association. However, results from the studies[42,43] with no TRIM effect could be confounding, as the patient involved received buffy-coat-depleted red blood cells.

In addition, there have been several trials as well as randomized controlled trials[41,44] investigating the association between allogenic blood transfusion and the risk of perioperative infection in which some of them showed an increased association between allogenic blood transfusion and perioperative infection, whereas, others refuted any such association. A recent Canadian experience[45] in cardiac surgery, hip fracture repair, and ICU patients, before and after implementation of universal leukoreduction, showed small but statistically significant differences in hospital mortality between the two study periods. The TRICC trial[46,47] by Hebert *et al*., in 1999, showed clearly that mortality and multiorgan dysfunction were higher in ICU patients who had been standardized to a liberal transfusion protocol compared to those who had been transfused using the conservative transfusion protocol. The recent hemovigilance report[48] from the French hemovigilence network clearly indicates that the incidence of bacterial sepsis and NHFTRs was significantly decreased (3.8 vs. 1.7% and 32.9 vs. 25.8%, respectively) following the implementation of universal leukoreduction.

## Recommended Leukodepletion Strategies for Developing Countries

### 

#### Modification of the component preparation technique

Adopting a buffy coat method of component preparation generally gives a log 1 leukoreduction and to a great extent can minimize the FNHTRs. A thorough quality practice can be achieved if leukodepletion is done in the top and bottom bags, with the help of certain dedicated equipments such as automatic component extractors. The blood components prepared with this method can give the desired therapeutic benefits to a majority of patients, even on regular transfusion therapy, where transfusions become troublesome due to repeated FNHTRs. However, the limitation of this technique is that it cannot prevent HLA-alloimmunization and CMV-transmission.

#### Selective pre-storage leukofiltration for patients on regular transfusions

The packed red cell selective pre-storage leukofiltration policy can be adopted for patients on regular transfusion therapy such as, thalassemia major patients, with skillful inventory management and active coordination between the transfusion therapy clinic and the blood bank.

Platelet concentrates can be pooled and leukofiltered for a select group of hemato-oncology patients in the blood centers, prior to the storage. This strategy can achieve 3-4 log leukoreduction, however, it requires dedicated technical manpower for stringent quality control and skillful inventory management, along with active coordination with the treating units.

#### Harvesting blood components through aphersis technology

Blood components harvested, through Apheresis technology, are generally 3-4 log leukoreduced, and provide better therapeutic benefits than the random donor products. This can be of great help to the patients' refractory to platelet transfusion with HLA alloimmunization, as the desired component can be harvested in sufficient quantity from an HLA-matched donor. However, the cost of the consumables act as a limiting factor for its utility at large, but it is the best option for those who can afford it. Mobilization of resources through philanthropic organizations for the benefit of poor patients should also be pursued.

## Conclusions

Thus, from the existing evidence, it is clear that leukoreduction is associated with reduced risk of some clinical conditions, but not for others. In some conditions, the evidence-based data is quite strong, but in others, the evidence-based criteria for instituting universal leukoreduction are not as strong. Therefore, the decision to implement universal leukoreduction should not be vested in only one clinical condition, particularly when the available evidence for that indication is not definitive. It is nonetheless clear that leukoreduction definitely reduces the rate of NHFTRs (level-I evidence); reduces CMV transmission (level I evidence); and is associated with the reduced risk of platelet refractoriness (level I evidence). With regard to the enhancement of tumor growth, the evidence is incomplete in humans and is only based on observational studies. Experimental animal studies have revealed that allogenic transfusions enhance tumor growth in animal models. The evidence for the decrease in the incidence of postoperative infection and multiorgan dysfunction is quite strong (level-I); especially in patients undergoing cardiac surgery.

Therefore, keeping in view all of the above, universal leukoreduction seems to be justified, but the cost involved in such an endeavor would be immense, especially as far as our country is concerned. Thus keeping in view the evidence and cost involved in universal leukoreduction, it is not practically feasible to implement this policy, especially in developing countries and other under-resourced nations. However, the following suggested strategies would act as practical guidelines.
